# Comparison of patient preferences for fecal immunochemical test or colonoscopy using the analytic hierarchy process

**DOI:** 10.1186/s12913-015-0841-0

**Published:** 2015-04-23

**Authors:** Yinghui Xu, Barcey T Levy, Jeanette M Daly, George R Bergus, Jeffrey C Dunkelberg

**Affiliations:** Department of Family Medicine, Roy J. and Lucille A. Carver College of Medicine, University of Iowa, Iowa City, IA 52242 USA; Department of Epidemiology, College of Public Health, University of Iowa, Iowa City, IA 52242 USA; Department of Internal Medicine, Roy J. and Lucille A. Carver College of Medicine, University of Iowa, Iowa City, IA 52242 USA

**Keywords:** Colorectal cancer screening, Analytic Hierarchy Process (AHP), Patient preference, Colonoscopy, Fecal immunochemical test

## Abstract

**Background:**

In average-risk individuals aged 50 to 75 years, there is no difference in life-years gained when comparing colonoscopy every 10 years vs. annual fecal immunochemical testing (FIT) for colorectal cancer screening. Little is known about the preferences of patients when they have experienced both tests.

**Methods:**

The study was conducted with 954 patients from the University of Iowa Hospital and Clinics during 2010 to 2011. Patients scheduled for a colonoscopy were asked to complete a FIT before the colonoscopy preparation. Following both tests, patients completed a questionnaire which was based on an analytic hierarchy process (AHP) decision-making model.

**Results:**

In the AHP analysis, the test accuracy was given the highest priority (0.457), followed by complications (0.321), and test preparation (0.223). Patients preferred colonoscopy (0.599) compared with FIT (0.401) when considering accuracy; preferred FIT (0.589) compared with colonoscopy (0.411) when considering avoiding complications; and preferred FIT (0.650) compared with colonoscopy (0.350) when considering test preparation. The overall aggregated priorities were 0.517 for FIT, and 0.483 for colonoscopy, indicating patients slightly preferred FIT over colonoscopy. Patients’ preferences were significantly different before and after provision of detailed information on test features (p < 0.0001).

**Conclusions:**

AHP analysis showed that patients slightly preferred FIT over colonoscopy. The information provided to patients strongly affected patient preference. Patients’ test preferences should be considered when ordering a colorectal cancer screening test.

## Background

Colorectal cancer (CRC) is the third leading cause of cancer death for both men and women in the United States [1]. Screening for colorectal cancer leads to approximately a 50% mortality reduction [2]. The American College of Physicians (ACP) recently reviewed multiple guidelines for colorectal cancer and recommended that all average-risk adults 50 years of age or older should be offered any of the following screening methods: fecal occult blood testing annually, sigmoidoscopy every five years, or colonoscopy every 10 years [3]. Although CRC screening rates have gradually increased in the past 10 years, one-third of adults 50 and older still are not up-to-date with CRC screenings [4]. Individual preferences for a certain screening test have been found to influence uptake in a CRC-screening program [5,6]. Incorporating patients’ preferences into the clinical decision-making process may be a way to improve screening rates.

Colonoscopy is the most popular screening test for colorectal cancer in the United States [7], being a gold standard for early detection and prevention of colorectal cancer, even though no studies have shown it to be superior to a sensitive fecal occult blood test in an average-risk population to reduce morbidity and mortality from CRC. The newer fecal immunochemical test (FIT) is more sensitive [8,9], and cost-effective than the guaiac test [10], but is underused in the United States [11]. There is no difference in life-years gained when comparing a screening strategy with colonoscopy every 10 years vs. an annual sensitive fecal occult blood test, such as a FIT [12]. However, in 2011, the Centers for Disease Control and Prevention (CDC) reported that only 11.8% of patients aged 50–75 completed fecal occult blood tests within the previous year [13]. If we are to improve CRC screening rates, it would be helpful to obtain insight into the patient preferences between colonoscopy and FIT.

Previous studies have described variations in patient preferences for specific CRC screening tests. These studies suggest individuals who value accuracy the most are more likely to select colonoscopy [14], whereas others preferred fecal occult blood testing because of its lower complication rate and simpler procedure [6,15]. Recently, several studies [16-19] have applied a method called the Analytic Hierarchy Process (AHP) to evaluate patient preferences for CRC screening tests. The unique contribution of this paper is that we compared patient preferences for FIT vs. colonoscopy after patients had completed both tests.

The purposes of this study were to: 1) analyze patient preferences for colorectal cancer screening between FIT and colonoscopy; 2) identify the test features that are important in the decision making; and 3) assess whether test preferences are associated with patients’ knowledge of the tests. It was anticipated that the information from this study would improve physician-patient discussions on screening options and enhance compliance with colorectal cancer screening.

## Methods

### Recruitment

The study and methods were approved by the University of Iowa Institutional Review Board and all participants provided written informed consent.

The study sample was comprised of patients scheduled for a colonoscopy at the University of Iowa Hospitals and Clinics. Patients were eligible for the study if they met the following criteria: 1) adults age 40 to 75 years, 2) scheduled for screening or surveillance colonoscopy, 3) colonoscopy scheduled within 10 days to 6 weeks, 4) valid address and telephone number, and 5) English speaking. Patients were excluded if they met any of the following: 1) familial polyposis syndromes, ulcerative colitis, or Crohn’s disease; 2) personal history of colon cancer; 3) active rectal bleeding; 4) change in bowel habits; or 5) pencil-like stools in the past two months.

Between January 22, 2010 and November 22, 2011, research staff in the Department of Family Medicine at the University of Iowa identified each patient with a scheduled colonoscopy from the electronic medical record system, Epic. A recruitment mailing (including a cover letter, two informed consents, a single-sample FIT kit, and a postage-paid return envelope) was sent to 2336 potential participants. Participants were asked to complete the FIT kit at home up to the day before they started their colonoscopy preparation and mail it to the study team. If the informed consent and FIT were returned, and the colonoscopy was completed, subjects were mailed a follow-up questionnaire the day after completing the colonoscopy. Non-responders were reminded by follow-up telephone calls and second mailings when necessary.

### Follow-up questionnaire

The follow-up questionnaire was developed by a group comprising practicing family physicians, including an expert in issues concerning medical decision-making (GB). The information and questions provided in the questionnaire were discussed and revised numerous times after deliberation. The information was based on the current CRC screening guidelines, literature, and the website of the Centers for Disease Control and Prevention. Participants were aware that if they completed a FIT and the result were positive, then a colonoscopy would be recommended (if they chose FIT as a screening in the “real” world). The final questionnaire developed by the research team consisted of 2 multiple-choice questions asking patient preference between FIT or colonoscopy; 6 questions related to the AHP model; 3 questions on preferences with various assumptions for out-of-pocket costs; 4 questions on personal and family history, and 7 questions on demographics. Patients were first asked which test they preferred based on the experience of having had both tests (FIT and colonoscopy). This question was a multiple-choice format to allow for patients to choose one test. Participants then answered the remaining questions based on the AHP model. The three main criteria (test accuracy; test complications; and test preparation, frequency, and procedure) were described in detail for each test. Participants then compared the relative importance between each possible pair of the three test features. They were asked to identify the more important test feature or whether they were equally important when selecting a colorectal cancer screening test. An example of questions follows:

Thinking about each pair of features, which feature is most important to you? Check one box.

- □ Accuracy of the test
- □ Avoiding complications
- □ Both are equally important

If they were not equally important, the participants were requested to choose one answer from the following 3 options:

How much more important?

- □ Not very much
- □ Somewhat
- □ Much more

The participants were then provided with side-by-side comparison information between FIT and colonoscopy for each of the three test features (test accuracy; test complications; and test preparation, frequency and procedure). Participants repeated the comparison process to determine their preferences with respect to each test feature. An example question follows:

Please indicate which test you prefer based on the information on the **accuracy** of the test we provided above. Check one box.

- □ FIT every year
- □ Colonoscopy every 10 years
- □ Either test is fine (no preference for either one)

If they preferred one test over the other, the participants were requested to choose one answer from the 3 options of “not very much”, “somewhat”, or “much more”.

After the questions on AHP model, we once again asked participants which tests they preferred after reading the detailed information on each test provided in the questionnaire. Patient personal and family history of digestive disease and demographic information such as age, gender, education, insurance, and income were also collected.

### Analytic Hierarchy Process (AHP)

AHP is a widely used decision-making method developed by Saaty [20,21] in the 1970s to assist people in making complex decisions. In the AHP model, the problem is decomposed into a hierarchy of goal, criteria and alternatives, with the goal placed at the top, criteria placed at the intermediate level and alternatives at the bottom. The elements in each hierarchical group are compared as pairs with respect to their importance in making the decision. These comparisons are used to obtain the proportional weights of the importance of the criteria and the relative importance of the alternatives in terms of each individual decision criterion. In the last step of the process, final priorities are calculated across the hierarchy for each of the decision alternatives. The alternative that attains the highest final priority is thought to be the most suitable decision.

**Figure 1:**
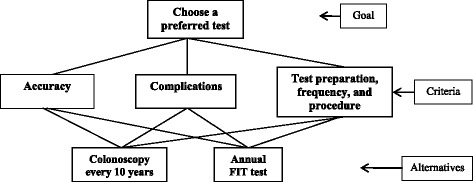
The structure of the Analytic Hierarchy Process model.

The model used for this study is shown in Figure 1. The goal of the decision, shown on the top, defined as “preferred test”, was to determine the patients’ preferences for colonoscopy or FIT for colorectal cancer screening. We focused on three test features related to decision-making in colorectal cancer screening: 1) test accuracy; 2) frequency of complications; and 3) preparation, frequency and procedure as the decision criteria for this problem. The alternatives were comprised of two of the recommended screening tests, colonoscopy every 10 years or an annual FIT test. The relative importance of three features was compared by constructing a 3 × 3 matrix of the three test features for the second level. The two alternatives were compared with respect to each of the test features as well, leading to three 2 × 2 matrices for the third level. The final step was to synthesize the results to obtain the final priorities of the two screening methods. We multiplied each alternative by the priority of its criterion and added the resulting weights for each alternative to calculate its final priority.

### AHP computations and data analysis

The collected data were analyzed with SAS software and the codes were written specifically for the AHP application (YX). The comparison of importance was converted to a numerical value using a scale of 1 to 4, where 1 meant equal importance (or no preference), 2 “not very much”, 3 “somewhat”, and 4 “much more important”. The geometric mean method was used to calculate an eigenvector for each patient, and the values of the weights were normalized so they summed to 1. A consistency ratio (CR) was calculated for each patient at the second level. If the CR value was greater than 0.15, the matrix was considered inconsistent and the patient’s judgments were excluded [18,22]. We did not calculate the CR at the third level, because for the matrices of size 2 × 2 the CR is not applicable, but we excluded patients’ judgments if they were excluded at the second level. Tables 1 and 2 shows a case of the input data and how the eigenvector for level 2 and final priorities were calculated. In this case, the highest weight criterion was “accuracy” (0.625), followed by “complications” (0.239), and “preparation” (0.136). The CR was low (0.0559) and it was considered to be a consistent judgment. The final priority of the given alternative was obtained from the weight of the alternative multiplied by the priority of each criterion. The final priorities of the alternatives were “FIT” (0.418) and “Colonoscopy” (0.582) for this participant, indicating that this patient preferred colonoscopy every 10 years more than a FIT annually.

**Table 1:** **A case of the pairwise comparison and priority calculation (consistency ratio (CR) = 0.0559)**

	**Accuracy**	**Complication**	**Preparation**	**Geometric means**	**Normalized priorities**
**Accuracy**	1	3	4	$\sqrt[3]{1\times 3\times 4}=2.289$	2.289/(2.289 + 0.874 + 0.5) = 0.625
**Complication**	1/3	1	2	0.874	0.239
**Preparation**	1/4	1/2	1	0.500	0.136

**Table 2:** **Calculations to obtain the final priorities**

	**Accuracy (0.625)**	**Complication (0.239)**	**Preparation (0.136)**	**Final priorities**
**FIT**	0.25	0.667	0.75	0.418
**Colonoscopy**	0.75	0.333	0.25	0.582

Final priority of FIT = 0.625 × 0.25 + 0.239 × 0.667 + 0.136 × 0.75 = 0.418.

Final priority of Colonoscopy = 0.625 × 0.75 + 0.239 × 0.333 + 0.136 × 0.25 = 0.582.

While participants acted as separate individuals, the aggregation of individual priorities was calculated with an arithmetic mean for synthesizing individual decisions into a group decision [23].

Demographic characteristics of participants who were included and excluded in the AHP analysis were summarized. A *t*-test was used to compare the means of the continuous variables, and the chi-square test was used to compare the percentages between two groups. To evaluate the changes of the patients’ preferences between FIT and colonoscopy before and after they read the detailed information in the questionnaire**,** the SAS FREQ procedure with the agreement test for symmetry was used for the comparison. All analyses were performed using SAS, version 9.3 (SAS Institute Inc, Cary, North Carolina).

## Results

**Figure 2:**
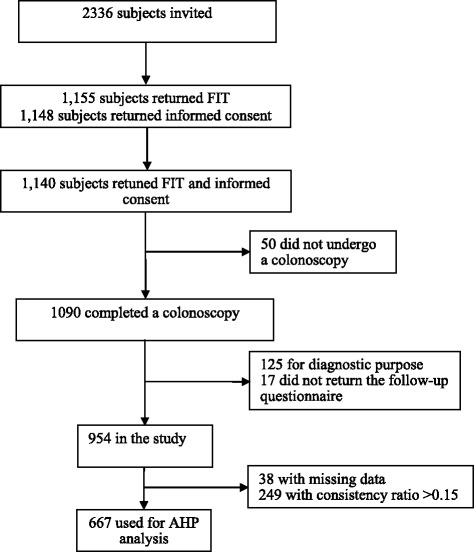
Participant enrollment flow chart.

As shown in Figure 2, of the potential 2336 patients, 1140 patients returned a consent form and a FIT (49%). A total of 1090 patients completed a colonoscopy; however, 136 were excluded from analysis for having a diagnostic colonoscopy and/or not returning follow-up questionnaire, leaving 954 patients in the study for analysis. Of the 954 patients, 667 patients (667/954 = 70%) were included in AHP analysis with consistency ratios (CR) ≤ 0.15. Patient characteristics of included and excluded patients in the AHP model are shown in Table 3. The age was significantly younger in the included patients than that in the excluded patient (56.7 vs 58.0, p = 0.014). There were no significant differences between included and excluded patients for other demographic characteristics including gender, marital status, race, ethnicity, education level, insurance status, income and location. Of the 667 patients included in the AHP analysis, the average time for returning the follow-up questionnaire after colonoscopy was 16.9 (SD 18.3) days. Of these 667 patients, 59% were female, 71% were married, 93% were white, and only 1% were Hispanic. Eighty-one percent had graduated from college or higher. Approximately 65% of patients reported annual household incomes of $40,000 or more, and most patients reported having some type of health insurance. Almost two thirds were from urban areas in Iowa.

**Table 3:** **Demographics in the included and excluded subjects (n=954)**

**Variable**	**Included subjects (n =667)**	**Excluded subjects (n =287)**	**p-values**
**Age in years (SD)**	56.7 (7.4)	58.0 (7.4)	0.0135
**Gender**			0.3476
Female	399 (59.9%)	162 (56.6%)	
Male	267 (40.1%)	124 (43.4%)	
**Marital Status**			0.6326
Single	166 (24.9%)	65 (22.8%)	
Married	468 (70.3%)	203 (71.2%)	
Widowed	32 (4.8%)	17 (6.0%)	
**Race**			0.9115
White	617 (93.1%)	268 (94.4%)	
Black	17 (2.6%)	6 (2.1%)	
Asian	17 (2.6%)	7 (2.5%)	
American Indian	3 (0.5%)	1 (0.4%)	
Others	9 (1.4%)	2 (0.7%)	
**Ethnicity**			0.6120
Hispanic	7 (1.1%)	4 (1.4%)	
**Education level**			0.1134
High school or less	112 (16.8%)	64 (22.3%)	
Some college or higher	552 (82.8%)	221 (77.0%)	
**Insurance status**			
Private	446 (67.2%)	186 (65.5%)	0.6161
Medicaid/Iowa Care	129 (19.4%)	59 (20.8%)	0.6338
Medicare	110 (16.6%)	53 (18.7%)	0.4334
None	12 (1.8%)	7 (2.4%)	0.5165
**Annual income**			0.7372
< $40,000	204 (30.6%)	88 (30.7%)	
$40,000 to < $80,000	169 (25.3%)	80 (27.8%)	
≥ $80,000	266 (39.9%)	105 (36.6%)	
Unreported	28 (4.2%)	14 (4.9%)	
**Rurality**			0.2249
Rural	217 (32.5%)	105 (36.6%)	
Urban	450 (67.5%)	182 (63.4%)	

Table 4 displays the average priorities for each criterion at level 2 and the average weights for the two alternatives with respect to each criterion at level 3. The final column shows the average of individual final priorities. The average priorities for the second level criteria of accuracy, complications, and test preparation were 0.457, 0.321, and 0.223, respectively, indicating that participants considered accuracy as the most important, followed by complications, and test preparation. Participants preferred colonoscopy (0.599) compared with FIT (0.401) with respect to accuracy; they preferred FIT (0.589) compared with colonoscopy (0.411) with respect to avoiding complications; and preferred FIT (0.650) compared to colonoscopy (0.350) with respect to test preparation. The average of individual final priorities for FIT was 0.517, and for colonoscopy was 0.483, indicating participants had a slight preference for FIT over colonoscopy overall.

**Table 4:** **Average local and final priorities based on AHP model (n=667)**

	**Accuracy**	**Complications**	**Preparation**	
	**Local priorities**			**Final priorities**
**Alternatives**	**0.457**	**0.321**	**0.223**	
**Colonoscopy**	0.599	0.411	0.350	0.483*
**FIT**	0.401	0.589	0.650	0.517*

*The final priorities were averages based on the individual final priorities.

Patients’ preferences from the multiple choice questions asked directly before and after the provision of detailed information are displayed in Table 5. Of the 382 subjects who initially preferred FIT, 222 (58.1%) continued to prefer FIT after information about the test, but 139 (36.4%) changed their preferences from FIT to colonoscopy. Among the 130 subjects initially preferring colonoscopy, 114 (87.7%) continued to prefer colonoscopy after information about the test, while only 7 (5.4%) changed their preferences from colonoscopy to FIT. Overall, 391 (61.5%) subjects kept their preferences the same. The kappa coefficient was 0.40 (95% CI, 0.34, 0.45), indicating fair agreement before and after the provision of information. The test for symmetry indicated that patients changed their preferences significantly following provision of detailed information (p < 0.0001). Patients’ preference priorities through the AHP model and from the directly-asked question after the provision of information were also compared. The kappa coefficient was 0.57 (95% CI, 0.51, 0.62), indicating moderate agreement between the two formats for obtaining preferences. The test for symmetry indicated that there were significant differences between the two formats for preferences (p < 0.0001).

**Table 5:** **Subjects’ preference for colon cancer screening before and after information provided (n=636)**

**Before information provided**	**After information provided**			
	**FIT every year**	**Colonoscopy every 10 years**	**Either test is fine**	
FIT every year	222	139	21	382 (60.1%)
Colonoscopy every 10 years	7	114	9	130 (20.4%)
Either test is fine	27	42	55	124 (19.5%)
	256 (40.3%)	295 (46.4%)	85 (13.4%)	636

Preferences were also elicited under hypothetical out-of-pocket costs. Under the assumption the same out-of-pocket costs for both tests, 43% preferred to be screened by FIT, colonoscopy was preferred by 42%, and 15% felt either test would be fine. When assuming an out-of-pocket cost of $40 for FIT and $4000 for colonoscopy, preferences changed significantly (p < 0.0001 by the test for symmetry): 77% preferred to be screened by FIT, colonoscopy was preferred by 17%, and 6% felt either test would be fine.

There was no significant association between patients’ preference priories and their race, marital status, insurance, or rural/urban residency. Females were more likely to prefer annual FIT than males, 54% vs 43%, respectively, p = 0.011. About 28% patients with an education of high school or less indicated equal priorities between annual FIT and colonoscopy compared to 11% for those with college or higher education, p < 0.01. The preference priorities also varied by household income. As household incomes increased, patients tended to prefer colonoscopy. The percent preferring colonoscopy for those household income less than $40,000, $40,000 to $80,000, and greater than $80,000 were 34%, 46%, and 51%, respectively, p < 0.01.

## Discussion

To our knowledge, this is the first study to measure preferences for CRC screening methods with patients who had immediate prior experience with both the colonoscopy and a fecal immunochemical test. Deciding among the screening options was a complex decision problem because each option presented advantages and disadvantages. In the present study, we used a large cohort of patients who were scheduled for colonoscopy. We demonstrated in the AHP model that patients value accuracy more highly than test complications and preparation. Overall, patients slightly preferred annual FIT over colonoscopy.

In this study, the decision-making process in the colonoscopy screening choices was conducted with a questionnaire based on AHP. The difficulty and confusion for patients to make a choice consistent with their values and preferences for colon cancer screening has been recognized [24]. AHP has the distinct advantage in that it decomposes a complex decision problem into a hierarchical structure of the criteria, which provides patients with a better focus on specific criteria and sub-criteria when determining priorities. It allows patients to make adequate judgments in a pairwise fashion, and the pairwise comparisons are straightforward and convenient. It provides a mechanism to check inconsistencies among the different pairwise comparisons by computing the consistency ratio. In our study using the AHP method, we not only quantified the relative importance of specific test features, but also computed relative weights for the alternatives in regard to each of the test features. Not surprisingly, there were some discrepancies in the preferences through the AHP model and from the directly-asked question. Patients answered the directly-asked questions intuitively. It is difficult for patients to make consistent decisions when faced with unfamiliar problems involving trade-offs between the advantages and disadvantages. The AHP was designed to help them make more informed choices in a step-by-step manner. On the other hand, investigators need sufficient knowledge and understanding of the problem being examined in order to successfully structure the AHP model and include all of the elements in the model with accuracy. The validity of the AHP process has been extensively tested in the well-designed models [25].

Our findings are consistent with other studies [5,26-30] that indicate a fairly close preference between colonoscopy and FIT as options for colorectal screening. However, other studies used qualitative ratings and ranking survey methods to determine the proportion of patients who prefer each of the CRC tests. A few studies [16-18] have used the AHP method to study preferences and priorities regarding colorectal cancer screening. Dolan et al. [17,18] indicated that patient priorities varied widely and cannot be predicted using demographic factors, numeracy, or literacy skills. Katsumura and colleagues [16] found that subjects gave higher priority to colonoscopy than to FOBT. However, this study was conducted in Japan among internet users, in a group of subjects who may have attained higher levels of education and annual household income. These studies illustrated the importance of identifying patients’ preferences in choosing among currently recommended colorectal cancer screening options. However, in these studies, subjects had not had the experience of completing both a colonoscopy and FIT, and our study is unique in that our questionnaires were conducted after subjects had experienced both tests and thus patients could factor their experience into their knowledge of the tests.

Previous studies consistently reported that colonoscopy and the stool blood test were the most often-preferred options by patients [5,30]. The number of colonoscopy procedures has risen steadily in the United States [13] because of its advantages, such as accuracy, sensitivity, allowing diagnosis and therapy in one session, and a longer interval between tests if no abnormal findings. The number of FOBTs performed annually decreased gradually from 21.1% to 11.8% between 2002 and 2010 [13]. Primary care physicians tend to recommend colonoscopy over other screening tests regardless of a patient’s preference. Hawley et al. [5] found 49% of the patients preferred to make medical decisions themselves, only 10% of the patients favored having a physician make their medical decisions. A recent study by Inadomi and colleagues [31] tested the patient-centered approach in 1000 patients who were randomized into three arms: FOBT only, colonoscopy only, or a choice of either test. Participants who were offered only colonoscopy completed the screening at a significantly lower rate (38%) than those who were offered FOBT (67%) or given a choice of either test (69%). This study indicates that not permitting a patient to choose their colorectal cancer screening test may contribute to non-compliance and potentially a screening failure. On the other hand, presenting multiple options for CRC screening may increase patient confusion and as a result, patients may fail to be screened [24]. The psychology literature has noted that too many choices constitute a barrier to decision-making and lead people simply to make no choice at all [32]. Our findings of the fairly close preference between colonoscopy and FIT have implications for clinicians that they could offer either of the tests and present the advantages and disadvantages of each when discussing CRC screening. Patients should be made aware of the advantages of FIT (sample collected in the privacy of one’s home, no dietary or other restrictions, no test preparation, no complications) and that no studies have shown colonoscopy is better than a sensitive fecal occult blood test for average risk individuals to reduce morbidity and mortality from CRC. A decision modeling analysis showed no difference in life-years gained between a strategy of annual fecal immunochemical testing and colonoscopy every 10 years [12]. In addition, many European countries, Great Britain, and Australia have a program of biennial fecal occult blood testing and reserve colonoscopy for those with positive fecal occult blood tests [33-36].

Our results confirmed that test preferences were associated with the importance of the test features determined by the patients. Our results using AHP analysis method were consistent with those studies that found patients valued accuracy as more important [27,37,38], and test preparation as the least important. In our study, when patients considered the accuracy, they were more inclined to select colonoscopy; when they considered test preparation or test complications, they favored FIT. It is important for physicians to identify a particular patient’s values regarding these test features, and to adopt a shared decision-making approach when discussing screening options.

Earlier studies [27,30] reported the association between race/ethnicity, education level, and income. Patients of non-Latino ethnicity, those with higher household incomes and higher education were more likely to prefer colonoscopy, but did not find association between test preferences and gender. We found that patient priorities differed by gender, education level, and household income. More women preferred FIT over colonoscopy and those with higher household incomes tend to prefer colonoscopy. Our results indicate that physicians should also consider individual patient’s demographic characteristics during the shared decision-making process.

In our study, we found that 30% patients provided inconsistent responses on the AHP questions. One possible reason that we had a relatively high percentage of patients with inconsistent responses was because all patients completed the questionnaire on paper and mailed them to us. We were not able to have the opportunity to provide feedback and correct substantially inconsistent judgments. This highlights the need to develop clinically feasible decision-support tools that can provide feedback for inconsistent responses to facilitate this decision-making process.

The comparison between patients’ preferences before and after the information was provided showed the significant impact that the information provided had on a patient’s preferred screening method. In the present study, the provision of the screening information changed the preferred screening method for 38% of the subjects. The percent of changing preference was low in the group which chose colonoscopy initially (12%) compared to the group which initially chose annual FIT (42%). The possible explanation could be that patients may learn that colonoscopy provides more accurate results with a longer test interval and hence switch their preferences to colonoscopy. Although patients had experience with both tests, and this experience provided them with an understanding of the testing procedures, it is likely that some patients still lack sufficient knowledge of the test accuracy, the testing interval, and possible complications. Previous studies have shown that patients should be well informed about test information and be involved in the decision-making process [16]. The provision of information in influencing an initial choice highlights the importance of educating patients with the strengths and limitations of each screening option.

This study has several limitations. We implemented our study at a Midwestern academic medical center using an advanced electronic medical record system; our findings may not apply to less structured settings. Our sample was limited to mostly well-educated, white patients with insurance who were scheduled for colonoscopy screening or surveillance. Future research in a more racially diverse area should be completed, since preferences may vary by educational and socioeconomic levels. Although we asked some questions concerning cost, our study did not include cost among the test features in the AHP model. Our results from the two hypothetical out-of-pocket cost questions were consistent with two previous studies [39,40] on CRC screening preferences that indicated that the out-of-pocket costs can affect preferences. Patients who value out-of-pocket expenses as an important factor in their decision-making might have been more inclined to choose the FIT. Our study offered the FIT at no cost to patients, and this might have influenced patient preferences toward FIT. We excluded the 30% of subjects whose responses were inconsistent, and subjects were significantly older in the group whose responses were excluded.

## Conclusions

In summary, we have found that average-risk patients who experienced both colonoscopy and FIT tests did not have a distinct preference between FIT and colonoscopy for screening. We confirmed that patients prioritized accuracy over other features and their preferences can be linked to test features. Primary care physicians should discuss with patients the risks and benefits of screening tests for colorectal cancer, even though it is not possible to discuss all aspects of screening in a busy clinic setting. The discussions could focus on comparing the main test features of FIT and colonoscopy. Additional research to determine the role of the AHP model on the healthcare decision making is necessary.

## Abbreviations

ACP: American College of Physicians
AHP: Analytic Hierarchy Process
CDC: Centers for Disease Control and Prevention
CR: Consistency ratio
CRC: Colorectal cancer
FIT: Fecal immunochemical test
